# Assays for quantification of male and female gametocytes in human blood by qRT-PCR in the absence of pure sex-specific gametocyte standards

**DOI:** 10.1186/s12936-020-03291-9

**Published:** 2020-06-23

**Authors:** Claire Y. T. Wang, Emma Ballard, Stacey Llewellyn, Louise Marquart, Teun Bousema, James S. McCarthy, Katharine A. Collins

**Affiliations:** 1 QPID Laboratory, Centre for Children’s Health Research, Brisbane, QLD Australia; 2 QIMR Berghofer Medical Research Institute, Brisbane, QLD Australia; 3 Radboud Institute for Health Science, Radboud University Medical Center, Nijmegen, The Netherlands

**Keywords:** Malaria, Gametocytes, VIS, CHMI, QRT-PCR, Droplet digital PCR, Transmission blocking, Pfs25, PfMGET

## Abstract

**Background:**

Malaria transmission from humans to *Anopheles* mosquitoes requires the presence of gametocytes in human peripheral circulation, and the dynamics of transmission are determined largely by the density and sex ratio of the gametocytes. Molecular methods are thus employed to measure gametocyte densities, particularly when assessing transmission epidemiology and the efficacy of transmission-blocking interventions. However, accurate quantification of male and female gametocytes with molecular methods requires pure male and female gametocytes as reference standards, which are not widely available.

**Methods:**

qRT-PCR assays were used to quantify levels of sex-specific mRNA transcripts in *Plasmodium falciparum* female and male gametocytes (*pfs25* and *pfMGET*, respectively) using synthetic complimentary RNA standards and in vitro cultured gametocytes. Assays were validated and assay performance was investigated in blood samples of clinical trial participants using these standards and compared to absolute quantification by droplet digital PCR (ddPCR).

**Results:**

The number of transcript copies per gametocyte were determined to be 279.3 (95% CI 253.5–307.6) for the female-specific transcript *pfs25*, and 12.5 (95% CI 10.6–14.9) for the male-specific transcript *pfMGET*. These numbers can be used to convert from transcript copies/mL to gametocyte/mL. The reportable range was determined to be 5.71 × 10^6^ to 5.71 female gametocytes/mL for *pfs25*, and 1.73 × 10^7^ to 1.73 × 10^1^ male gametocytes/mL for *pfMGET.* The limit of detection was 3.9 (95% CI 2.5–8.2) female gametocytes/mL for *pfs25*, and 26.9 (95% CI 19.3–51.7) male gametocytes/mL for *PfMGET*. Both assays showed minimal intra-assay and inter-assay variability with coefficient of variation < 3%. No cross-reactivity was observed in both assays in uninfected human blood samples. Comparison of results from ddPCR to qRT-PCR assays on clinical blood samples indicated a high-level agreement (ICC = 0.998 for *pfs25* and 0.995 for *pfMGET*).

**Conclusions:**

This study reports the validation of qRT-PCR assays that are able to accurately quantify female and male *P. falciparum* gametocytes at sub-microscopic densities. The assays showed excellent reproducibility, sensitivity, precision, specificity, and accuracy. The methodology will enable the estimation of gametocyte density in the absence of pure female and male gametocyte standards, and will facilitate clinical trials and epidemiological studies.

## Background

Successful transmission of *Plasmodium* infection from humans to mosquitoes requires that a female *Anopheles* mosquito imbibes at least one mature male and one mature female gametocyte in a blood meal. The density and the ratio of female to male gametocytes in peripheral blood are therefore key determinants of infectiousness to mosquitoes, the dynamics of transmission, transmission epidemiology in endemic settings, and for evaluating the efficacy of transmission blocking interventions such as drugs and vaccines [1–6]. Thus, techniques to accurately detect and quantify male and female gametocyte densities are required [7].

Successful transmission has been reported to occur at sub-microscopic gametocyte levels both in endemic areas during asymptomatic infections, and in volunteer infection studies (VIS), also referred to as controlled human malaria infection (CHMI)-transmission studies [6, 8, 9]. Understanding the contribution of sub-microscopic infections to the human infectious reservoir would greatly assist elimination efforts. Gametocyte densities have traditionally been measured by thick or thin film microscopy; however, both methods have limited sensitivity and cannot accurately measure gametocyte densities below 10,000 gametocytes per mL [10]. To overcome the limited sensitivity of microscopy, quantitative reverse transcription PCR (qRT-PCR) and quantitative nucleic acid sequence based amplification (NASBA) assays targeting gametocyte specific mRNA transcripts have been developed [6, 11–15]. The highly conserved *pfs25* gene transcript is present in abundance in female gametocytes and is, therefore, commonly targeted for quantification by qRT-PCR [7, 14]. More recently, male-specific gametocyte mRNA transcripts have been described [5, 16, 17], and among them *pfMGET* (Pf3D7_1469900) shows an abundant transcription profile [5] making it an appropriate candidate for quantitation of male gametocytes. The use of both female- and male-specific assays allows evaluation of the gametocyte sex ratio, a parameter of interest in quantifying infectivity to mosquitoes, particularly at lower gametocyte densities [5, 18–21]. The *Pfs25*-*PfMGET* combination for sex ratio determination was recently validated by immunofluorescence assays on field samples from gametocyte donors [22]. This ratio may be particularly important for assessment of gametocytocidal drug activity, as the less abundant male gametocytes may be more readily sterilized or killed by some anti-malarial drugs [14, 23, 24].

qRT-PCR methods have superior sensitivity over microscopy allowing the quantification of gametocyte densities across the epidemiologically relevant range [1]. These assays require the use of reference standards to generate standard curves for quantification. These ideally consist of biological reference standards, namely pure populations of female and male gametocytes. However, preparation of such material requires laborious approaches to culture and FACS-sort gametocyte reporter lines that are not readily available to all laboratories [25]. An alternative method for generation of standard curve material is the use of synthetic standards such as cRNA, which can be prepared by any laboratory. Although this approach permits quantification of the absolute number of mRNA transcripts, the assay does not allow the quantification of gametocyte numbers unless the number of copies of the target mRNA transcript expressed per gametocyte are known. As the number of mRNA transcripts expressed per gametocyte differs for each target [5, 25], the expression level of each must be known in order to convert transcripts per mL to gametocytes per mL. By determining these conversion factors it would significantly improve the assessment of gametocyte density and sex ratios by qRT-PCR using synthetic cRNA standards.

This study describes the validation of two qRT-PCR assays that enable the accurate and sensitive detection and quantification of female (*pfs25*) [11] and male (*pfMGET*) [5, 6] gametocytes in whole blood samples without the need for purified female and male gametocytes reference standards.

## Methods

### RNA extraction

250 µL of packed red blood cells (pRBCs) from each clinical trial participant (see below) were stored (1:5) in 1250 µL RNA Protect Cell Reagent (Qiagen, Australia) at − 80 °C until RNA extraction. Prior to RNA extraction, 5 µL of equine arteritis virus (EAV) culture (which gives a Cq of ~ 30) was spiked into each sample as an internal control to monitor extraction efficiency and qRT-PCR inhibition as detailed below [26]. RNA extraction was performed on the whole sample using RNeasy Plus Mini Kit (Qiagen, Australia) following manufacturer’s instructions with treatment of DNase on-column digestion using RNase-Free DNase set (Qiagen, Australia) to eliminate genomic DNA. 100 µL of RNA extract was eluted.

### Female and male gametocyte qRT-PCR assays

Sex-specific qRT-PCR assays were used to measure RNA transcripts specific to female and male gametocytes (Table 1). The previously described female marker assay was designed to target the *Plasmodium falciparum* gametocyte surface protein (*pfs25*) mRNA (Genbank accession number AF154117) [11]. The male marker assay targeted the exon–exon junction of the *P. falciparum* PF3D7_1469900 mRNA transcript (Genbank accession number XM_001348805) recently characterized as male gametocyte-enriched transcript (*pfMGET*) [5, 6].

**Table 1:** List of oligonucleotides and synthetic cRNA used in this study

Oligo/synthetic cRNA name	Sequences	Target	References
*pfs25* (female gametocytes)	Forward: 5′-AAATCCCGTTTCATACGCTTGTAA-3′ Reverse: 5′-CAGTTTTAACAGGATTGCTTGTATCTAATATAC-3′ Probe: 5′-FAM-ACCAAATGAATGTAAGAATGTAACTTGTGGTAACGGT-BHQ1-3′	*P. falciparum pfs25* mRNA	[11]
*pfMGET* (male gametocytes)	Forward: 5′-AAAATTCGGTCCAAATATAAAATCCTG-3′ Reverse: 5′-CTTCATCAATTAAAAATCCCTTTTTTGT-3′ Probe: 5′-FAM-CCTGGTAAAAAACAGCTCCAGCA-BHQ1-3′	*P. falciparum* PF14_0063 (PF3D7_1469900) mRNA	[5, 6]
EAV (extraction control)	Forward: 5′-GGCGACAGCCTACAAGCTACA-3′ Reverse: 5′-CGGCATCTGCAGTGAGTGA-3′ Probe: 5′-FAM-TTGCGGACCCGCATCTGACCAA-BHQ1-3′	EAV ORF7	[26]
*pfs25* cDNA with T7	5′-TAATACGACTCACTATAGGGAACAAATCCCGTTTCATACGCTTGTAAATGTAATCTTGGATATGATATGGTAAATAATGTTTGTATACCAAATGAATGTAAGAATGTAACTTGTGGTAACGGTAAATGTATATTAGATACAAGCAATCCTGTTAAAACTGTTT-3′		This study
*pfMGET* cDNA with T7	5′-TAATACGACTCACTATAGGGAACAAAATTCGGTCCAAATATAAAATCCTGTTCAGAAGAAAAAAAAAGTATCCTGGTAAAAAACAGCTCCAGCATTAAAAACACAAAAAAGGGATTTTTAATTGATGAAGTTT-3′		This study

All qRT-PCR assays were conducted with One-Step RT-PCR mix (Qiagen, Australia) using methods previously described [6, 11] with 0.45 µM of each primer and 0.18 µM of Taqman probe in each PCR reaction. Amplification was performed in a Rotorgene 6000 or Q instrument (Qiagen, Australia) under the following cycling conditions: 50 °C reverse transcription for 30 min, 95 °C incubation for 15 min, followed by 45 cycles of 95 °C for 15 s and 60 °C for 60 s. Additional PCR reactions with heat inactivated reverse transcriptase were included to ensure genomic DNA was undetected in each qRT-PCR reaction. RNA extraction efficiency was monitored through the use of EAV RT-PCR assay (Table 1) [6, 26]. From each batch of extractions the mean C_q_ ± 2 standard deviations (SD) of EAV samples were calculated and used as the acceptable range for extraction quality. If EAV C_q_ values fell outside this range, extraction was considered inhibited and a repeat extraction and PCR was performed. For each batch, mean C_q_ values typically range from 28 to 30 with an SD of ~ 0.5 (SD values exceeding 1 suggest highly variable extraction efficiency in that batch).

### Synthetic complementary RNA (cRNA) standard preparation and standard curve construction

Standard curves were generated from serial-diluted synthetic cRNA controls. Synthetic linear double stranded DNA fragment containing qRT-PCR target sequences with T7 promoter (Thermo Fisher Scientific, Australia) (Table 1) was in vitro transcribed to cRNA using HiScribe™ T7 High Yield RNA Synthesis Kit (New England BioLabs) following the manufacturer’s instructions, and then subjected to two cycles of DNase digestion (RNase-free DNase set, Qiagen, Australia) to eliminate synthetic DNA contamination. cRNA was then purified using the Qiagen RNeasy mini kit (Qiagen, Australia). Quantity (copy numbers) of neat cRNA control was calculated based on molecular weight measured by the High Sensitivity RNA Qubit assay (Thermo Fisher Scientific) [6]. Seven concentrations of the cRNA standards were prepared by making tenfold serial dilutions (*pfs25*: 1.59 × 10^6^ to 1.59 copies/µL*; pfMGET*: 2.16 × 10^5^ to 2.16 × 10^−1^ copies/µL) in uninfected human whole blood extracts to reflect similar matrix specimen type to clinical trial samples. These seven concentrations constitute the cRNA standard curves for each assay and were run in replicates (n = 6) to generate an ‘external standard curve’ for each assay. Aliquots of the seven concentrations for each standard curve were also run with every qRT-PCR assay in singlet during clinical trials. The highest concentration standard in each assay run was used as a calibrator for quantification using the external standard curves. This was done by importing the linear regression model from the external standard curve (*pfs25* slope: − 3.395, *pfMGET* slope: − 3.236) into all qRT-PCR runs within Rotorgene software with fixing the intercept to the C_q_ value of the highest concentration standard to calculate RNA copies in test samples. The remaining six standards acted as positive controls, and were used for confirming adequate PCR efficiency (> 90%). Long-term monitoring of drift and stability of cRNA standards was conducted using Levey-Jennings plots (acceptable range set to mean Cq ± 2SD).

### Assay sensitivity

To determine limit of detection (LOD) and intra-assay variability neat cRNA was serial-diluted in uninfected human blood extracts to produce 14 concentrations of *pfs25* cRNA (1.59 × 10^6^ to 0.05 copies/µL) and 11 concentrations of *pfMGET* cRNA (2.16 × 10^5^ to 0.07 copies/µL), with emphasis on closer dilutions in the lower range to ensure enough data points for accurate estimation. Replicates (n = 5–8) of each dilution and a negative control (uninfected human blood extracts) were analysed on separate qRT-PCR runs on separate days (6 for *pfs25* and 3 for *pfMGET*) on a Rotorgene Q instrument (Qiagen, Australia) with the same batch of mastermix used for each assay. The experiments and validation of the *pfs25* assay occurred before repeating the process with the *pfMGET* assay. These data sets are hereafter referred to as data set A (*pfs25*) and data set B (*pfMGET*).

### Assay specificity

Analytical specificity was determined by comparing the sequence of the nucleic acid target (primer and probe sequences) to sequences available on publicly accessible databases using BLAST search tool (NCBI) to check its specificity to *P. falciparum pfs25* and PF3D7_1469900 (*pfMGET*) transcripts. No cross reactions were observed for other malarial mRNA transcripts and human genomic DNA or mRNA transcripts. Blood from malaria-naïve volunteers enrolled in clinical trials (*pfs25* n = 66, *pfMGET* n = 11) was assessed for assay specificity.

### Female and male gametocyte ddPCR

Since no “gold standards” were available for RNA quantification, ddPCR was utilized to generate absolute quantification of RNA copy numbers to confirm the accuracy of RNA estimates in both cRNA standards and infected blood. The female and male gametocyte qRT-PCR assays were adapted to the droplet digital PCR (RT-ddPCR) format. cRNA controls and RNA extracts from participants (see below) were analysed on QX200 ddPCR system (BioRad, Australia). The RT-ddPCR reactions were prepared using One-Step RT-ddPCR Advanced Kit for Probes (BioRad, Australia). Each 20 µL of ddPCR reaction contained 5 µL of 1× One-Step RT-ddPCR Supermix, 0.45 µM of each primer, 0.18 µM of Taqman probe, and 5 µL of RNA template. The RT-ddPCR mix was loaded onto the QX-200 AutoDG automated droplet generator (BioRad, Australia) to partition the reaction into nanolitre-sized droplets before being transferred to a 96 well plate for thermal cycling. Amplification was performed on a C1000 Touch thermal cycler (BioRad, Australia) with the following conditions: reverse transcription at 50 °C for 1 h, enzyme activation at 95 °C for 10 min, 40 cycles of 95 °C for 30 s and 60 °C for 1 min with ramp rate settings to 2 °C/s, with final step of enzyme deactivation at 98 °C for 10 min and 12 °C forever. Amplification products were read on the QX-200 Droplet Reader (BioRad, Australia) and quantification was determined using the associated QuantaSoft analysis software (BioRad, Australia). Reactions containing uninfected human blood extracts were used to determine the negative amplitude threshold for quantification analysis of samples. Quantification results by ddPCR were compared to the results by qRT-PCR.

### Female and male gametocyte reporter lines

Gametocytes were cultured and maintained using the PfDynGFP/PfP47mCherry reporter line as previously described [5, 25], and were sorted using the Coulter Epics Elite flow cytometer (Beckman Coulter) or the BD FACS Aria SORP flow cytometer keeping cells at 4 °C in SA buffer at Radboud University (Nijmegen, Netherlands) [25]. The sorted female and male gametocytes (10^6^ gametocytes/mL) were stored in RNA Protect Cell Reagent and then frozen (Qiagen). Gametocytes were thawed and RNA extracted using methods described above. A tenfold dilution series of the extracted RNA was made ranging from 10^6^ to 10 female gametocytes/mL and 10^5^ to 1 male gametocytes/mL for calculation of the conversion factor for each assay. The male and female dilution series were run in duplicate on 3 separate occasions (a total of 36 PCR reactions per assay using aliquots of the same dilution series). The *pfs25* and *pfMGET* mRNA transcript numbers were determined in these samples using the qRT-PCR assays with cRNA standards. These data sets are hereafter referred to as data set C (*pfs25*) and data set D (*pfMGET*). The study design was a randomized block design with the dilution series as treatments, daily runs as blocks and technical replicates used to estimate intra-assay variability.

### Gametocyte positive human blood samples

Gametocyte positive human blood samples (*pfs25* n = 33, *pfMGET* n = 36) were obtained from previously reported CHMI-transmission studies. These studies were single-centre, open-label clinical trials run concurrently between 2015 and 2016 at Q-Pharm Pty Ltd (Brisbane, Australia) [6]. Participants were healthy, malaria-naïve adults aged between 18 and 55 years. Participants were inoculated with ~ 2800 parasite infected RBCs on day 0 and were treated with 480 mg piperaquine 7 or 8 days after inoculation to clear asexual parasitaemia. Whole blood samples were collected before and after piperaquine treatment and were analysed by female- and male- specific qRT-PCR and subsequently ddPCR for verification of quantification as described above. These clinical trials were approved by the QIMR Berghofer Medical Research Institute Human Research Ethics Committee, and all participants gave written informed consent before inclusion in the study. The clinical trials are registered with ClinicalTrials.gov (NCT02431637 and NCT02431650). This data set is hereafter referred to as data set E.

### Statistical analysis

Statistical analysis of the conversion factors for both assays, reportable range, precision and sensitivity was conducted in SPSS version 22 (IBM Corp, Armonk, NY). Data sets A and B (described above) were used to assess linearity, calculate the LOD_95%_ and intra-assay variability, and validate the reportable range. The LOD_95%_ is defined as the concentration at which cRNA template can be consistently detected in 95% or more of samples with acceptable precision as indicated by the 95% confidence interval and was calculated using Probit regression. The intra-assay SD for each concentration was calculated using analysis of variance accounting for day using all technical replicate data. The overall SD is the pooled SD estimates across all concentrations. The reportable range is the set of standards used for the final qRT-PCR assays during clinical trials and the limits at which the calibration curve can be used to accurately and precisely estimate copy numbers while preserving the linearity of estimation from the regression equation used for statistical calibration was validated here. A linear regression model was used to estimate the relationship between log_10_ concentration of standard and C_q_ value. For this model technical replicates were averaged within days. Only the data for concentrations within the range of 5.71 × 10^6^ to 5.71 female gametocytes/mL for the *pfs25* assay and 1.73 ×  × 10^7^ to 1.73 × 10^1^ male gametocytes/mL for the *pfMGET* assay were used in the linear regression analysis. The slope estimate was used to calculate qPCR efficiency for the reportable range of gametocyte densities.

The inter-assay variability was determined using standard curve data from historical records of 17 runs of 7 study cohorts for *pfs25* and 29 runs from 15 study cohorts for *pfMGET*. These data sets are hereafter referred to as data set F (*pfs25*) and data set G (*pfMGET*). The inter-assay SD for each concentration was calculated using analysis of variance accounting for study cohort. The overall SD is the pooled SD estimates across concentrations, $s_{pooled} = \sqrt {\frac{{\left( {n_{1} - 1} \right)s_{1}^{2} + \left( {n_{2} - 1} \right)s_{2}^{2} \cdots + \left( {n_{k} - 1} \right)s_{k}^{2} }}{{n_{1} + n_{2} + \cdots n_{k} - k}}}$. Relative variability was measured as the percent coefficient of variation (%CV) of C_q_ for each standard concentration. A linear regression model was used to estimate the relationship between log_10_ concentration of standard and C_q_ value separately for each standard curve. The slope estimate was used to calculate qPCR efficiency with the range of qPCR efficiencies over clinical trials and mean value given for each assay.

The conversion factors for each assay to convert from copies/mL to gametocytes/mL were calculated using data sets C and D (detailed above). An analysis of variance on the residual difference in log_10_ copies per gametocyte between log_10_ copies/mL and log_10_ gametocytes/mL accounting for day, sample (concentration) and their interaction was used to obtain the mean for each assay conversion factor. The 95% confidence intervals (CI) were calculated using the t distribution and relevant degrees of freedom. The inter-assay SD is the square root of the mean squares value for the interaction term. The intra-assay SD is the square root of the mean squared error.

Accuracy was assessed using intraclass correlation coefficient (ICC), paired *t* test and Passing-Bablok regression to examine differences in log_10_ gametocytes/mL of whole blood between qRT-PCR and ddPCR (data set E) using R Studio (ver. 1.1.442, R version 3.4.4). The reference standard was ddPCR in these analyses.

## Results

### Estimating transcript numbers per gametocyte using purified in vitro cultured female and male gametocytes

Two dilution series of purified female and male gametocytes of known concentration obtained from the *PfDyn*GFP/*PfP47*mCherry reporter line were analysed by qRT-PCR (data sets C and D), and used to calculate the number of copies of each mRNA target expressed per gametocyte for converting copies/mL to gametocytes/mL. The conversion factors were determined to be 279.3 copies/female gametocyte (95% CI 253.5–307.6) for the female-specific marker *pfs25* and 12.5 copies/male gametocyte (95% CI 10.6–14.9) for the male-specific marker *pfMGET.* The pooled inter-assay SD of the log_10_ copies/gametocyte was calculated to be 0.105 (*pfs25*) and 0.180 (*pfMGET*). The pooled intra-assay SD of the log_10_ copies/gametocyte was calculated to be 0.044 (*pfs25)* and 0.080 (*pfMGET*).

### Female and male gametocyte qRT-PCR assay performance

Sex-specific qRT-PCR assays were validated to quantify mRNA transcript levels specific to female (*pfs25*) and male (*pfMGET*) gametocytes. Both assays showed reliable amplification across a large linear range with good precision, sensitivity and specificity. The linearity, accuracy and precision of the reportable range, (5.71 × 10^6^ to 5.71 female gametocytes/mL for *pfs25*, and 1.73 × 10^7^ to 1.73 × 10^1^ male gametocytes/mL for *pfMGET*) was validated. Template was also detected at 0.18 gametocytes/mL for *pfs25* and to 5.59 gametocytes/mL for *pfMGET*, however standards below the reportable range showed increasing variability amongst the samples detected and some loss of linearity which would result in inaccurate quantification below the reportable range. The amplification efficiency over the reportable range was 96.8% for *pfs25* and 98.1% for *pfMGET*. No amplification was observed in the negative control extracts (*pfs25* n = 33, *pfMGET* n = 6) from uninfected human blood extracts.

The LOD_95%_ for these assays was determined to be 3.9 female gametocytes/mL of whole blood (95% CI 2.5–8.2) for *pfs25,* and 26.9 male gametocytes/mL of whole blood (95% CI 19.3–51.7) for *pfMGET* when a starting volume of 250 µL of packed RBCs (equivalent to 500 µL whole blood) was analysed.

There was a tendency for both intra- and inter-assay variability to increase at lower dilutions but the %CV of the C_q_ was less than 3% for each standard in both assays (for both inter- and intra-assay variation). The overall intra-assay SD was 0.52 C_q_ units for *pfs25* and 0.51 C_q_ units for *pfMGET*, indicating minimal variability between replicates (Tables 2 and 3; data sets A + B). The overall inter-assay SD was 0.50 C_q_ units for *pfs25* and 0.46 C_q_ units for *pfMGET* (Tables 4 and 5; data sets F + G), which indicates minimal variability between qRT-PCR runs.

**Table 2:** *pfs25* (female gametocyte) qRT-PCR assay for assessing intra-assay variability between technical replicates

Standard (gametocytes/mL)	Detected (% positive)	Mean C_q_ (SD)	Range C_q_	%CV C_q_
5.71 × 10^6^	36/36 (100.0%)	17.14 (0.19)	16.67–17.62	1.1
5.71 × 10^5^	34/34 (100.0%)	20.53 (0.23)	19.87–20.96	1.1
5.71 × 10^4^	35/35 (100.0%)	23.94 (0.18)	23.60–24.33	0.7
5.71 × 10^3^	36/36 (100.0%)	27.13 (0.19)	26.57–27.49	0.7
5.71 × 10^2^	36/36 (100.0%)	30.57 (0.22)	30.09–31.29	0.7
5.71 × 10^1^	36/36 (100.0%)	33.87 (0.25)	33.10–34.50	0.8
1.15 × 10^1^	21/21 (100.0%)	36.15 (0.45)	35.36–36.94	1.2
7.16	21/21 (100.0%)	37.26 (0.55)	36.40–38.87	1.5
5.71	14/15 (93.3%)	38.09 (0.92)	36.81–39.65	2.4
2.86	18/21 (85.7%)	38.58 (1.09)	37.23–40.96	2.8
1.43	16/21 (76.2%)	39.77 (1.01)	38.43–42.32	2.5
0.72	32/42 (76.2%)	40.07 (0.80)	38.92–41.8	2.0
0.36	8/21 (38.1%)	40.42 (0.80)	38.99–42.17	2.0
0.18	3/20 (15.0%)	40.51 (n/a^a^)	40.43–40.56	n/a
Negative control	0/33	Not detected		
Total	346/395 (87.6%)	33.14 (0.52^b^)	16.67–42.32	

C_q_: quantification cycle, SD: standard deviation, %CV: percent coefficient of variation for detected samples, n/a: not applicable, Negative control: uninfected human blood control

^a^Could not be calculated due to one positive sample per day

^b^Overall SD

**Table 3:** *pfMGET* (male gametocyte) qRT-PCR assay for assessing intra-assay variability between technical replicates

Standard (gametocytes/mL)	Detected (% positive)	Mean C_q_ (SD)	Range C_q_	%CV C_q_
1.73 × 10^7^	15/15 (100.0%)	16.56 (0.06)	16.44–16.70	0.4
1.73 × 10^6^	15/15 (100.0%)	19.86 (0.06)	19.70–20.08	0.3
1.73 × 10^5^	15/15 (100.0%)	23.19 (0.06)	23.03–23.32	0.3
1.73 × 10^4^	15/15 (100.0%)	26.57 (0.10)	26.41–26.76	0.4
1.73 × 10^3^	15/15 (100.0%)	29.94 (0.20)	29.68–30.52	0.7
1.73 × 10^2^	14/14 (100.0%)	33.28 (0.39)	32.63–33.93	1.2
86.19	24/24 (100.0%)	34.35 (0.40)	33.32–35.08	1.2
43.10	24/24 (100.0%)	35.47 (0.92)	34.09–37.13	2.6
21.55	22/24 (91.7%)	36.47 (0.75)	34.94–37.25	2.1
11.17	14/24 (58.3%)	36.59 (0.49)	35.47–37.21	1.3
5.59	8/24 (33.3%)	36.73 (0.64)	35.89–37.47	1.7
Negative control	0/6	Not detected		
Total	181/209 (86.6%)	30.34 (0.51^b^)	16.44–37.47	

C_q_: quantification cycle, SD: standard deviation, %CV: percent coefficient of variation, Negative control: uninfected human blood control

^b^Overall SD

**Table 4:** *Pfs25* (female gametocyte) qRT-PCR assay for assessing inter-assay variability between runs for 7 clinical study cohorts

Standard (gametocytes/mL)	n	Mean C_q_ (SD)	Range C_q_	%CV C_q_
5.71 × 10^6^	17	16.73 (0.27)	15.66–17.43	1.6
5.71 × 10^5^	17	19.93 (0.36)	18.71–20.82	1.8
5.71 × 10^4^	17	23.32 (0.31)	22.47–24.63	1.3
5.71 × 10^3^	17	26.60 (0.45)	25.12–27.87	1.7
5.71 × 10^2^	24	30.04 (0.45)	29.07–31.21	1.5
5.71 × 10^1^	11	34.02 (0.38)	32.94–34.97	1.1
5.71 × 10^0^	13^a^	37.14 (0.87)	35.79–38.42	2.3
Total	116	26.29 (0.50^b^)	15.66–38.42	

C_q_: quantification cycle, SD: standard deviation, %CV: percent coefficient of variation

^a^Overall SD

^b^Out of 16 runs

**Table 5:** *pfMGET* (male gametocyte) qRT-PCR assay for assessing inter-assay variability between runs for 15 clinical study cohorts

Standard (gametocytes/mL)	n	Mean C_q_ (SD)	Range C_q_	%CV C_q_
1.73 × 10^7^	28	16.42 (0.26)	15.76–17.57	1.6
1.73 × 10^6^	29	19.68 (0.28)	19.11–20.90	1.4
1.73 × 10^5^	29	22.97 (0.28)	22.32–24.13	1.2
1.73 × 10^4^	29	26.32 (0.33)	25.65–28.07	1.3
1.73 × 10^3^	29	29.71 (0.34)	28.85–31.30	1.2
1.73 × 10^2^	29	33.08 (0.64)	32.11–35.42	1.9
1.73 × 10^1^	21^a^	36.30 (0.90)	34.57–37.67	2.5
Total	194	26.00 (0.46^b^)	15.76–37.67	

C_q_: quantification cycle, SD: standard deviation, %CV: percent coefficient of variation

^a^Out of 29 runs

^b^Overall SD

The range of PCR efficiency values calculated for all of the *pfs25* assay standard curves (data set F) run during clinical trials was 91 to 105% with a mean of 97%. The range of PCR efficiency values calculated for all of the *pfMGET* assay standard curves (data set G) was 93% to 107% with a mean of 100%.

No off target primer or probe interactions were identified by search of GenBank (BLAST, NCBI) indicating good analytical specificity of the two assays. Diagnostic specificity for both *pfs25* and *pfMGET* was demonstrated using blood samples from malaria-naïve individuals (*pfs25* n = 66, *pfMGET* n = 11), all being negative, to give a specificity of 100% with lower bounds of 95% confidence of 95.6% and 76.2%, respectively.

### Absolute quantification by ddPCR verifies qRT-PCR quantification

**Fig. 1:**
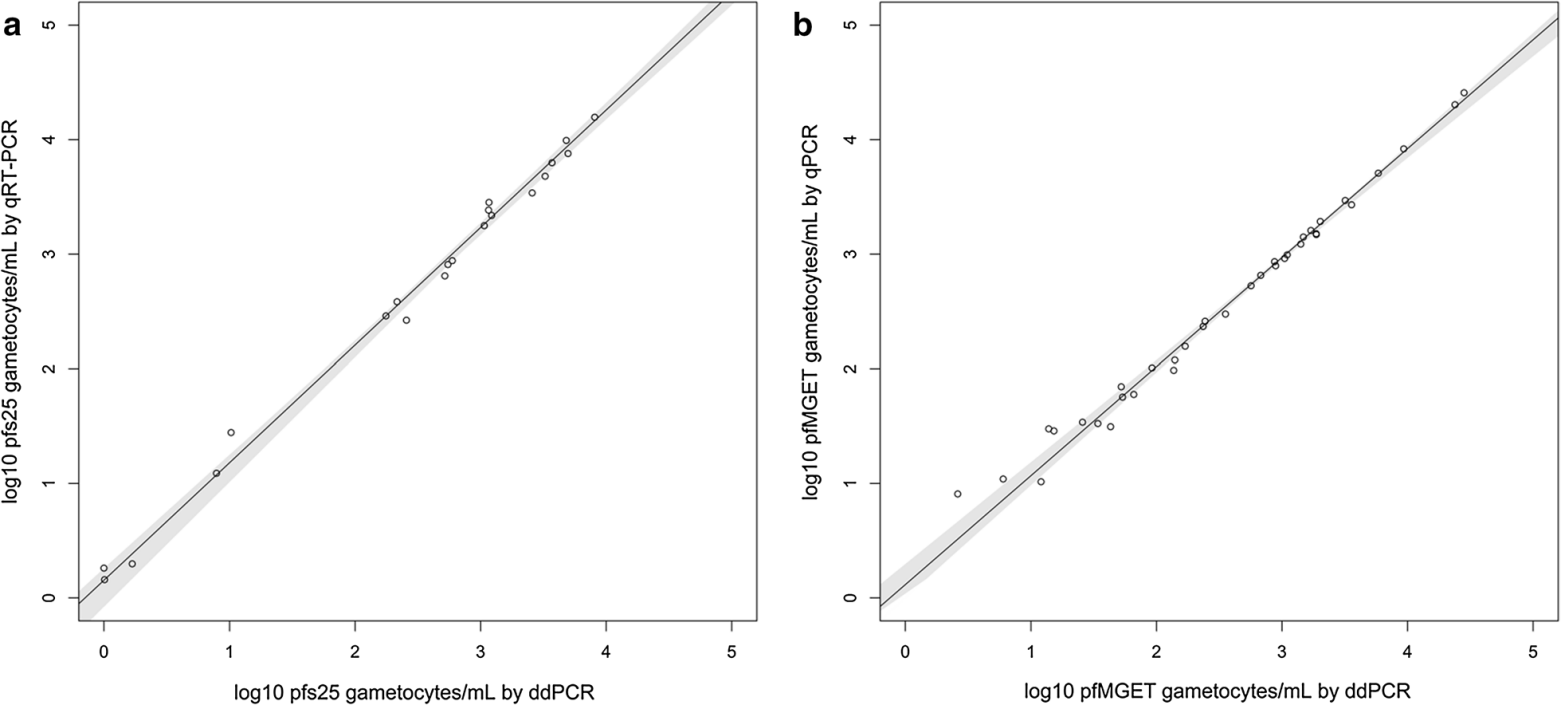
The plots show log_10_ gametocytes/mL by ddPCR and qRT-PCR. The solid black line represents the fitted Passing–Bablok regression line. The 95% confidence bounds, in grey, were calculated using the bootstrap quantile method. The female *pfs25* assay is shown to the left and the male *pfMGET* assay is shown to the right. The reference standard was ddPCR

To confirm the accuracy of the gametocyte-specific qRT-PCR assays, gametocyte-positive blood samples from two clinical trials were used to compare the quantitative results of the qRT-PCR assays to those from droplet digital PCR technology. Only samples with gametocyte densities greater than 1 gametocyte/mL as measured by qRT-PCR were included in the analysis for *pfs25* (n = 21) and *pfMGET* (n = 36). ICC values were very high, 0.998 (95% CI 0.996–0.999) for *pfs25* and 0.995 (95% CI 0.990–0.998) for *pfMGET*, indicating excellent consistency between methods. Overall qRT-PCR had higher mean log_10_ female gametocytes/mL values than droplet digital PCR (mean difference 0.21, 95% CI 0.17–0.26) but values for *pfMGET* were similar (mean difference 0.01 log_10_ male gametocytes/mL, 95% CI − 0.04–0.05). There was no evidence of non-linearity by Passing-Bablok regression nor of a systematic difference for each qRT-PCR assay (*pfs25* intercept 0.15 [95% CI − 0.08–0.26], *pfMGET* intercept 0.12 [95% CI 0.00–0.30]) (Fig. 1). There was no evidence of a proportional difference for *pfs25* (slope 1.03 [95% CI 0.99–1.09]); however, there was evidence of a slight proportional difference for *pfMGET* between methods at lower concentrations (below 2 log_10_ gametocytes/mL) (slope 0.95 [95% CI 0.89–0.98]).

## Discussion

A good understanding of the infectious reservoir of malaria and malaria transmission dynamics is required to inform the development and implementation of transmission-blocking interventions. To determine the relative contribution of sub-microscopic gametocyte densities to transmission [8, 9], molecular assays able to accurately detect very low gametocyte densities have been developed [5, 6, 15]. This study reports the validation of qRT-PCR assays for female and male gametocytes using cRNA standards. Use of these standards overcomes previous limitations of qRT-PCR assays that required purified and well-characterized female and male gametocyte reference materials to generate standard curves. To enable interpretation of such qRT-PCR data, the mean copy number of each transcript per gametocyte has been determined (279.3 for *pfs25 a*nd 12.5 for *pfMGET*). These figures are very similar to the copy numbers per gametocyte described by Meerstein-Kessel et al. who used a different batch of the same transgenic NF54 parasite line, (*pfs25* 231.7 (95% CI 199.1–269.8) and *pfMGET* 9.8 [95% CI 8.9–10.2)] [20] and to those estimated from in vivo samples (589 *pfs25* and 9.8 *pfMGET*) [6]. These estimates vary slightly between studies, likely due to different assay methodologies and statistical methods used to determine the conversion factors. However, reassuringly, despite these differences the estimates determined here are within the same range and remarkably similar considering the resolution that can be obtained with PCR. For example, the twofold difference in copy numbers between in vivo and in vitro estimates (588 to 279.3) correlates to less than a 1 Cq difference, which can commonly be observed in PCR in technical replicates of the same sample.

The two assays were validated and shown to be highly sensitive with their LOD_95%_ for female gametocytes determined to be 3.9 gametocytes/mL, and 26.9 gametocytes/mL for male gametocytes. In addition, the specificity and reproducibility of these two assays was confirmed. A significant advantage of these assays is that they are sufficiently sensitive to quantify sub-microscopic female and male gametocyte levels and determine gametocyte sex ratios in natural infections or during CHMI-transmission studies. The accuracy of these calculations is supported by the fact that absolute quantification of male and female gametocytes in clinical samples using ddPCR were very close to those derived from qRT-PCR assays. Quantification of log_10_ gametocytes/mL by qRT-PCR and ddPCR showed good agreement with little to no evidence of systematic or proportional bias by Passing Bablok regression. The paired t-test indicated that on average the log_10_ gametocytes/mL for ddPCR was 0.21 log_10_ gametocytes/mL lower than qRT-PCR for the female assay however the systematic difference by Passing Bablok, which is the median of Y_i_ − _1__i_, was 0.15 with a confidence interval including 0, indicating that there is a small but non-significant difference between the methods across the range of values. This supports the estimates of transcript abundance determined here, as well as demonstrating that qRT-PCR is sufficiently accurate where ddPCR is not available.

Determining the numbers of transcripts per gametocyte is challenging due to the need for pure populations of both female and male gametocytes. These samples are difficult to obtain from natural infections due to the presence of both gametocyte sexes and ring-stage parasites in peripheral circulation during most infections. Pure gametocyte samples can be obtained from in vitro cultured transgenic parasites; however, it is not currently possible to efficiently separate the female and male populations from wild type parasite cultures. Therefore, in this study a previously described transgenic NF54 parasite line, *PfDyn*GFP/*PfP47*mCherry, was used to generate gametocytes in vitro [5, 25]. Gametocytes produced using this line have fluorescent markers in the female and male gametocytes enabling purification of the two populations by FACS. Although this is currently the best method available for generating pure populations of female and male gametocytes, there are a number of limitations. Firstly, the levels of transcription of the two chosen targets may vary with gametocyte age, as seen in before with *pfs25* being predominantly expressed in stage V gametocytes [27]. Although a stage-V gametocyte culture was used here, low-level contamination of different stage gametocytes cannot be ruled out and could potentially result in a slight underestimation in copy numbers per gametocyte. In addition, it is plausible that transcript levels for *pfs25* and *pfMGET* may differ between in vivo and in vitro generated gametocytes or even during a single in vivo infection due to the differences and changes in local growth environments, as seen for other *Plasmodium* transcripts (recently reviewed by Llora-Batle et al. [28]), though this has not been reported for *pfs25* and *pfMGET* before. Likewise, it is unknown if there are differences in levels of these two transcripts in different strains of *P. falciparum*. These possible variations in numbers of transcript copies per gametocyte could therefore result in under of over estimation of gametocyte densities. However, the same caveats are not specific to using conversion factors and would also apply to all qRT-PCR assays that rely on mRNA transcript quantification. Reassuringly, in support of the calculations presented, the copy numbers per gametocyte estimated here were very similar to those estimated from in vivo samples during a CHMI-transmission study [6].

## Conclusions

This study presents the validation of *P. falciparum* male and female gametocyte-specific qRT-PCR assays that can quantify gametocyte densities with excellent reproducibility, sensitivity, specificity and accuracy. Moreover, sex-specific mRNA transcript levels per gametocyte were determined, enabling accurate quantification of gametocyte densities in the absence of pure female and male gametocyte standards. The methodology described here can enable the wider use of qRT-PCR assays for detecting and quantifying gametocyte densities over a broad range, including sub-microscopic gametocytes. This will facilitate studies that either evaluate transmission-blocking interventions, or aim to improve understanding of the infectious reservoir, which will be increasingly more valuable as malaria elimination gets closer.

## Data Availability

The data that support the findings of this study are available from the corresponding author upon reasonable request.

## Abbreviations

CHMI: Controlled human malaria infection
CI: Confidence interval
Cq: Quantification cycle
cRNA: Complementary RNA
CV: Coefficient of variation
ddPCR: Droplet digital PCR
EAV: Equine arteritis virus
ICC: Intraclass correlation coefficient
FACS: Fluorescent activated cell sorting
LOD: Limit of detection
mRNA: Messenger RNA
pRBCs: Packed red blood cells
qRT-PCR: Quantitative reverse-transcriptase PCR
SD: Standard deviation
TBIs: Transmission-blocking interventions
VIS: Volunteer infection study

## References

[1] Churcher TS, Bousema T, Walker M, Drakeley C, Schneider P, Ouedraogo AL (2013). Predicting mosquito infection from *Plasmodium falciparum* gametocyte density and estimating the reservoir of infection. Elife..

[2] Akim NI, Drakeley C, Kingo T, Simon B, Senkoro K, Sauerwein RW (2000). Dynamics of *P. falciparum* gametocytemia in symptomatic patients in an area of intense perennial transmission in Tanzania. Am J Trop Med Hyg.

[3] Jeffery GM, Eyles DE (1955). Infectivity to mosquitoes of *Plasmodium falciparum* as related to gametocyte density and duration of infection. Am J Trop Med Hyg.

[4] Bonnet S, Gouagna LC, Paul RE, Safeukui I, Meunier JY, Boudin C (2003). Estimation of malaria transmission from humans to mosquitoes in two neighbouring villages in south Cameroon: evaluation and comparison of several indices. Trans R Soc Trop Med Hyg.

[5] Stone W, Sawa P, Lanke K, Rijpma S, Oriango R, Nyaurah M (2017). A molecular assay to quantify male and female *Plasmodium falciparum* gametocytes: results from 2 randomized controlled trials using primaquine for gametocyte clearance. J Infect Dis.

[6] Collins KA, Wang CY, Adams M, Mitchell H, Rampton M, Elliott S (2018). A controlled human malaria infection model enabling evaluation of transmission-blocking interventions. J Clin Invest..

[7] Pett H, Goncalves BP, Dicko A, Nebie I, Tiono AB, Lanke K (2016). Comparison of molecular quantification of *Plasmodium falciparum* gametocytes by Pfs25 qRT-PCR and QT-NASBA in relation to mosquito infectivity. Malar J..

[8] Ouedraogo AL, Bousema T, Schneider P, de Vlas SJ, Ilboudo-Sanogo E, Cuzin-Ouattara N (2009). Substantial contribution of submicroscopical *Plasmodium falciparum* gametocyte carriage to the infectious reservoir in an area of seasonal transmission. PLoS ONE.

[9] Bousema T, Okell L, Felger I, Drakeley C (2014). Asymptomatic malaria infections: detectability, transmissibility and public health relevance. Nat Rev Microbiol.

[10] Karl S, Laman M, Koleala T, Ibam C, Kasian B, N’Drewei N (2014). Comparison of three methods for detection of gametocytes in Melanesian children treated for uncomplicated malaria. Malar J..

[11] Pasay CJ, Rockett R, Sekuloski S, Griffin P, Marquart L, Peatey C (2016). Piperaquine monotherapy of drug-susceptible *Plasmodium falciparum* infection results in rapid clearance of parasitemia but is followed by the appearance of gametocytemia. J Infect Dis.

[12] Schneider P, Schoone G, Schallig H, Verhage D, Telgt D, Eling W (2004). Quantification of *Plasmodium falciparum* gametocytes in differential stages of development by quantitative nucleic acid sequence-based amplification. Mol Biochem Parasitol.

[13] Niederwieser I, Felger I, Beck HP (2000). *Plasmodium falciparum*: expression of gametocyte-specific genes in monolayer cultures and malaria-positive blood samples. Exp Parasitol.

[14] Koepfli C, Yan G (2018). *Plasmodium* gametocytes in field studies: do we measure commitment to transmission or detectability?. Trends Parasitol..

[15] Hanron AE, Billman ZP, Seilie AM, Olsen TM, Fishbaugher M, Chang M (2017). Multiplex, DNase-free one-step reverse transcription PCR for *Plasmodium* 18S rRNA and spliced gametocyte-specific mRNAs. Malar J..

[16] Schneider P, Reece SE, van Schaijk BC, Bousema T, Lanke KH, Meaden CS (2015). Quantification of female and male *Plasmodium falciparum* gametocytes by reverse transcriptase quantitative PCR. Mol Biochem Parasitol.

[17] Santolamazza F, Avellino P, Siciliano G, Yao FA, Lombardo F, Ouedraogo JB (2017). Detection of *Plasmodium falciparum* male and female gametocytes and determination of parasite sex ratio in human endemic populations by novel, cheap and robust RTqPCR assays. Malar J..

[18] Meibalan E, Marti M (2017). Biology of malaria transmission. Cold Spring Harb Perspect Med..

[19] Bradley J, Stone W, Da DF, Morlais I, Dicko A, Cohuet A (2018). Predicting the likelihood and intensity of mosquito infection from sex specific *Plasmodium falciparum* gametocyte density. Elife..

[20] Meerstein-Kessel L, Andolina C, Carrio E, Mahamar A, Sawa P, Diawara H (2018). A multiplex assay for the sensitive detection and quantification of male and female *Plasmodium falciparum* gametocytes. Malar J..

[21] Schneider P, Greischar MA, Birget PLG, Repton C, Mideo N, Reece SE (2018). Adaptive plasticity in the gametocyte conversion rate of malaria parasites. PLoS Pathog.

[22] Gruenberg M, Hofmann NE, Nate E, Karl S, Robinson LJ, Lanke K (2020). qRT-PCR versus IFA-based quantification of male and female gametocytes in low-density *Plasmodium falciparum* infections and their relevance for transmission. J Infect Dis.

[23] Delves MJ, Ruecker A, Straschil U, Lelievre J, Marques S, Lopez-Barragan MJ (2013). Male and female *Plasmodium falciparum* mature gametocytes show different responses to antimalarial drugs. Antimicrob Agents Chemother.

[24] White NJ, Ashley EA, Recht J, Delves MJ, Ruecker A, Smithuis FM (2014). Assessment of therapeutic responses to gametocytocidal drugs in *Plasmodium falciparum* malaria. Malar J..

[25] Lasonder E, Rijpma SR, van Schaijk BC, Hoeijmakers WA, Kensche PR, Gresnigt MS (2016). Integrated transcriptomic and proteomic analyses of *P. falciparum* gametocytes: molecular insight into sex-specific processes and translational repression. Nucleic Acids Res.

[26] Balasuriya UB, Leutenegger CM, Topol JB, McCollum WH, Timoney PJ, MacLachlan NJ (2002). Detection of equine arteritis virus by real-time TaqMan reverse transcription-PCR assay. J Virol Methods.

[27] Farid R, Dixon MW, Tilley L, McCarthy JS (2017). Initiation of gametocytogenesis at very low parasite density in *Plasmodium falciparum* infection. J Infect Dis.

[28] Llora-Batlle O, Tinto-Font E, Cortes A (2019). Transcriptional variation in malaria parasites: why and how. Brief Funct Genomics..

